# Effect of green value capture on the manufacturing firm performance considering green dynamic capabilities and profitability models

**DOI:** 10.1371/journal.pone.0291773

**Published:** 2023-11-03

**Authors:** Baohong Li, Fansheng Meng, Shi Yin, Xiufen Wen

**Affiliations:** 1 School of Economics and Management, Harbin Engineering University, Harbin, China; 2 School of Economics and Management, Harbin Normal University, Harbin, China; 3 College of Economics and Management, Hebei Agricultural University, Baoding, China; 4 School of Management, Lanzhou University of Finance and Economics, Lanzhou, China; Universiti Utara Malaysia, MALAYSIA

## Abstract

Ensuring and improving the green value acquisition of manufacturing enterprises is one of the critical issues to be solved for manufacturing enterprises to achieve green value and high-quality development. Based on the theory of enterprise value acquisition and dynamic capability theory, we explain the "black box" of the relationship between green value creation and enterprise performance of manufacturing enterprises. The logical thread of "green value creation—profit model—enterprise performance" is constructed, and a green value acquisition mechanism model is proposed. Based on 263 questionnaires from Chinese manufacturing enterprises, the model is empirically tested using multiple regression analysis. The results show that green value creation has a positive impact on corporate performance, while the profit model plays a mediating role between green value creation and corporate performance. Green dynamic capability plays a positive moderating role between green value creation and corporate performance, while green active ability plays a positive moderating role between value creation and profit model.

## 1 Introduction

Environmental problems are serious and a consensus has been reached on green development. As the pillar of our national economy, manufacturing enterprises have long contributed to economic growth and environmental pollution. With the proposed dual carbon target, the pressure of green development of traditional manufacturing enterprises is prominent, and it is urgent to achieve high-quality development [1]. How to transform the green external economy into the green internal economy and how to encourage enterprises to carry out green innovation and green manufacturing has become one of the important issues to be investigated. Whether green practice can bring competitive advantage and economic benefits to manufacturing enterprises is often ignored in the literature [2].

Value creation is the most difficult and attractive problem facing any company. Value creation refers to the value created, only the potential value. Value acquisition is the value perceived and paid for by the consumer, i.e. the realized value. Value creation is about how to create more value. Value acquisition is about how to ensure value exclusivity, the ultimate direction of value movement, and how to allocate value to maximise the benefits of the company itself. At present, the way of value acquisition is difficult to adapt to the complex market environment, the difficulty of value acquisition is increasing, and the literature lacks the causal path of value creation and value acquisition [3]. The research on value creation and value acquisition based on the green practice situation of Chinese manufacturing enterprises has attracted the attention of scholars, the paper explains the mechanism of profit model and green dynamic capability on the green time of manufacturing enterprises, and puts forward some countermeasures and suggestions.

This article collects data through a survey questionnaire, verifies the hypothesis in the research model through regression analysis, and explores the mechanism of green value acquisition. This study expands the research path of green value acquisition mechanism in theory, and more comprehensively explains the path of green value creation to improve enterprise performance; in practice, relevant countermeasures have been provided for the green development of manufacturing enterprises; this provides a reference path for manufacturing enterprises to achieve green transformation and enhance green capabilities.

The rest of this article is organized as follows. Section 2 is the theoretical assumptions. Section 3 is methodology. Section 4 is the empirical analysis. Section 5 is the research results and discussion. Section 6 is the conclusion. Section 7 is the study limitation and future research.

## 2 Theoretical assumptions

Through relevant literature search and collation, it is found that there are relatively few relatively mature theoretical models in terms of green value acquisition mechanisms. According to Jing and Bao (2012), in the process of green value realization, green value transmission channels are built around customer needs, leading to green consumption and satisfaction of customer needs, as well as the realization of environmental and social values [4]. There is no in-depth research on the issue of green value creation and acquisition, and the research content is more focused on theoretical deduction. Based on a cross-case analysis of electric vehicles in China, Chen et al. (2015) proposed key mechanisms for value capture in the industrial ecosystem, arguing for guidelines within the industrial system, dedicated intellectual property rights, and ensuring understanding and communication among participants regarding business goals and needs [5]. Li and Zuo (2018) constructed a conceptual model of intellectual property value capture based on value capture theory and through a case study approach to provide theoretical support for the development of innovation ecosystems in high-tech industries [6]. And these value acquisition models have not yet conducted in-depth research on green value acquisition.

Green dynamic capability refers to a company’s ability to promote economic and environmental benefits and sustainable competitiveness through functional changes in the face of government environmental regulations, green market demand pressure, and sustainable expectations of stakeholders. It is the ability to perceive and capture business opportunities and value through government environmental regulations and consumer demand preferences. In addition, dynamic capabilities also can change the way resources are used and deal with new values. For example, environmental insight capability is mainly the ability of companies to understand the governed environment policy east of industry technology changes and consumer green demand forecast. Organizational flexibility capability can find appropriate communication channels, increase work flexibility, prompt innovation and organizational growth, digital capability plays a key role in the communication efficiency and the degree of cooperation and innovation advancement between different departments of a company, between a company and its stakeholders, and between a company and its customers. In conclusion, green dynamic capability refers to the unique ability of manufacturing enterprises to achieve sustainable development, adapt to government environmental regulations, and meet customers’ green needs by acquiring and integrating resources and technologies within the value network through digital technology, breaking through the original path dependency and forming the operation mechanism of the value network to achieve the harmony and unity of the value network and ecological environment with manufacturing enterprises as the core. Research in foreign countries is mainly focused on the impact of green concept green strategy on the performance of companies between the mechanisms [6–9].

### 2.1 Green value creation and corporate performance

Based on the deep excavation of customers’ green demand information and the tracking of government environmental policies, manufacturing companies identify more green value space and collaborate with stakeholders in R&D, production, sales, and recycling activities to produce green products and provide green services for customers [10]. The main idea of green value creation activities of manufacturing companies is to provide customers with the desired green products or services, i.e., new value and valuable experiences. When customers get new value from green products or services, it will stimulate their desire to buy products and services, which in turn improves the company’s recognition in the green market and gains more market share, thus promoting the improvement of enterprise performance. In this process, manufacturing enterprises reduce energy consumption and protect the environment through the research and development, introduction, and application of green technology; the promotion of digital technology lowers transaction costs; and the introduction of servitization reduces the energy consumption of enterprises. The reduction of cost inevitably leads to the improvement of manufacturing enterprises’ performance. Fang and Zhang (2014) explored the relationship between value creation and enterprise performance by taking the ecological business model of creative industries as a research object [11]. Liu et al. (2017) used business model innovation as the research object and found that the value creation dimension significantly improved firm performance [12]. Wang (2021) took the innovation ecosystem as the research object and examined that innovation ecosystem value creation and value acquisition, as two subsystems, interact with each other to drive the system evolution jointly and have an essential role in driving the value creation mode and the speed of value creation [13].

According to John Elkington, president of sustainability, the triple bottom line model of green value capture in manufacturing companies captures three aspects of corporate performance: environmental performance, economic performance, and customer value. Many scholars have deeply explored environmental performance based on environmental strategy, green innovation, and servitization perspectives. Environmental performance results from a firm’s environmental strategy and its implementation. For example, Chi et al. (2016) found that the preemptive environmental strategy positively impacts the firm’s environmental and economic performance [14]. Under the green innovation perspective, scholars have proposed that environmental performance is one of the results of green innovation activities and the specific components of environmental performance. For example, Zhu and Geng (2006) proposed that the environmental performance of a green supply chain of manufacturing enterprises includes six aspects: reduction of exhaust gas emissions, reduction of generated wastewater, reduction of solid waste, reduction of the use of hazardous/harmful toxic materials, reduction of frequency of environmental accidents, and improvement of environmental conditions of enterprises [15]. Yina and Fei (2011) concluded that corporate green innovation practices significantly and positively affect environmental performance [16].

According to Huang (2016), environmental performance is expressed as the effective reduction of pollutant generation and emission through green innovation, which reduces the pollution and threat to the ecological environment from process activities and promotes ecological improvement [17]. From the servitization perspective, Reiskin et al. (2000) found that the servitization of manufacturing helps to reduce the negative impact of physical elements on the environment, which in turn improves environmental performance [18]. Xu and Zhang (2021) found the servitization of manufacturing significantly enhances the green welfare effect under the dual perspective of pollution improvement and environmental total factor productivity [19]. Song and Zhang (2022) proposed paths to enhance environmental performance improvement based on the servitization of manufacturing enterprises. The first integrates service elements to promote enterprises’ green technology innovation. Second, the increase of input servitization rate by enterprises helps them to improve the efficiency of factor use, which in turn improves the environmental performance [20].

The primary issue for the survival and development of manufacturing enterprises is the pursuit of profitability, i.e., gaining economic benefits. By implementing green innovation, green production, green marketing, and other green value creation activities, manufacturing enterprises obtain lower operating costs, increased market share, and new green market opportunities, etc., and they will actively invest in the next round of green value creation activities and form a virtuous cycle. That is, the economic benefits brought by green value creation by manufacturing enterprises can meet enterprises’ production and development needs before they are motivated to implement green behaviors [21].

Economic performance revolves around cost reduction and increases in benefits or market opportunities. With cost reduction as the core of the discussion, for example, Zhu and Geng (2006) proposed to measure the economic performance of a green supply chain through four dimensions of material reuse and recycling to reduce procurement cost, energy consumption cost reduction, waste treatment cost reduction, and waste emission cost reduction [15]. On the other hand, Dong and Ying (2010) measured the economic benefits of ecosystems in two dimensions: direct and indirect economic benefits [22]. Kexin et al. (2013) proposed from the perspective of green process innovation that the economic performance in green process innovation is mainly reflected in two aspects: on the one hand, the improvement of green manufacturing capability of enterprises, the increase of the proportion of green technologies, products or services in the market and the proportion of green technologies, products or services in the total output value; on the other hand, the reduction of pollutant generation and emission, the comprehensive utilization expenditure of three wastes and On the other hand, the reduction of pollutant generation and emission, the reduction of expenditure on complete utilization of three wastes and environmental pollution treatment, and the reduction of production cost [23]. Incorporating economic performance with profits and market opportunities, for example, Shi et al. (2019) suggested that economic performance includes cost reduction and profit increase [24]. Yang et al. (2022) pointed out that green innovation leads to the growth of economic benefits, such as reduced environmental costs, reduced future environmental liabilities, increased market share and new market opportunities, and an excellent environmental image [25].

Customer value refers to the total value provided by manufacturing companies to customers that contains green and low carbon. It includes green product value, green service value, green image value, and other content. Customer value creation is an important part of the value-creation process and is the inevitable and reasonable result of enterprises’ continuous search for competitive advantage.

With the shift from a product-led to a customer-led model, customers are involved in product development, production, and sales. Therefore, customer value has begun to be paid attention to and included in the study of firm performance. Prahalad and Pamaswany (2004) emphasized the importance of interaction between customers and firms, arguing that customer value is the value of customer experience and that the interaction process between firms and customers’ value can be created [26]. Holbrook (2006) defined customer value as an experience based on interaction with relative preferences and argued that customer-perceived value mainly includes economic, hedonic, social, and spiritual values [27].

Richard (2007) argued that users are an essential source of enterprise value, that user experience is the key to measuring the magnitude of value creation, and that firms can create value by improving user value benefits [28]. The application of digital technology promotes the evolution of value activity carriers from value chains to value networks and ecosystems. An and Wu (2020) proposed that customer value is the core of value networks, and enterprises need to continuously innovate product technology to meet the dynamic needs of customers [29]. The value creation process of each participating entity in the value network revolves around customer value, and it coordinates the enterprises in the network to assist each other and create value together through the resources mobilized by the core enterprise.

H1: Green value creation significantly and positively affects corporate performance.

H1a: Green value creation significantly and positively affects environmental performance.

H1b: Green value creation significantly and positively affects economic performance.

H1c: Green value creation significantly and positively affects customer value.

### 2.2 The mediating role of the profit model

The profitability model is a strategic intention of a firm to create differences in various ways and is essential for the firm to achieve its current revenue goals [30]. Schweizer (2005) proposed that a firm’s revenue source and potential is one of the components of a business model [31]. Itami and Nishino (2010) viewed value creation and value capture as inseparable components and distinguished between the two. The profitability model reflects the value capture mechanism and is how a firm earns profits while delivering value to customers [32].

Scholars have studied the components of profitability models from several perspectives. Zhang and Wang (2010) proposed that the profitability model includes a firm’s cost structure, revenue sources, and revenue potential [30]. Jing and Bao (2012) suggested that the profitability model consists of the enterprise’s cost structure and revenue sources. In the cost structure, green value creation is life-cycle cost management based on customers’ green demand, and it will significantly reduce customer costs. Among the revenue sources, service-oriented green value creation expands the traditional revenue methods, such as the customer pay-as-you-go approach, consulting services with added value, etc. [4]. Jane (2018) proposed that the widespread use of the Internet has reduced the transaction costs between firms, and the frequency of transactions has increased. Firms use their products as a medium to continuously provide functional services to customers, which in turn creates continuous and stable revenue [33]. As digital technologies emerge and spread their applications, traditional revenue models are being innovated. Scholars have conducted relevant studies on revenue model innovation in profitability models. Revenue model innovation refers to generating new profit sources by providing new products and services and introducing new pricing models [34], and Wang (2011) summarized five revenue model innovation paths from the perspective of network reconstruction, which are portfolio value concession, additional and value-added product innovation, customer segmentation, third-party market introduction, and revenue source reversal [35].

Scholars’ research focuses on enterprise value capture, which provides a reference for in-depth analysis of green value capture in manufacturing enterprises. Due to the specificity of green value capture activities in manufacturing firms, combining customer needs, government environmental regulations, and other specific contexts is necessary to design profitability models. Profitability, as a way to capture value, refers to how an enterprise generates revenue, reduces costs, and earns profits, answering how an enterprise effectively captures economic returns [36]. Manufacturing enterprises ’ green production behavior and green services change market regulations, and enterprises have to reconstruct or innovate profit models, such as new customer relationships, access to current or potential customer markets, and the accumulation and application of green knowledge and technology in implementing green behavior. Therefore, the way of green value acquisition for manufacturing enterprises—the profit model differs from other enterprises’ profit models in that the profit model should be conducive to attracting customers to buy a green business or enjoy green services. In addition to meeting the functions of customers’ traditional products, it also needs to produce no harm to the environment or greatly reduce the degree of harm to the environment. Therefore, manufacturing companies’ innovative profitability, locking users, and establishing isolation mechanisms will increase customers’ demand for green products or services. Manufacturing companies achieve their own environmental and economic performance while realizing customers’ green needs.

First, to maintain long-term access to stable revenue and profitability levels remain high, companies must not only build diversified revenue sources but also combine customers’ consumption patterns and demand conditions. Digitization, the advancement of service in manufacturing companies, allows companies to meet consumers’ personalized needs. Zhang et al. (2022) proposed that digitalization has prompted enterprises to adopt innovative revenue models, and digital representation, connection, and aggregation processes break through traditional profit models, such as the free model, to enhance the linkage between enterprises and customers to achieve reciprocity between information, technology and value creation, and to expand corporate value acquisition channels [37]. The initial investment cost of green value creation activities of manufacturing enterprises is huge, and it is not easy to make short-term profits. Therefore, the innovation of the profitability model is particularly important. Second, the time lag of high initial investment and profit creation of manufacturing enterprises’ greening requires transforming enterprises to have strong financing ability and bear high financial leverage risk [38], which requires relationship resources between enterprises and financial institutions as their profit model resources. The service-oriented transformation of capital-intensive manufacturing enterprises emphasizes financing capability, which is closely related to resources such as brand image, financial strength, and external relationships. Manufacturing enterprises with stronger financing ability can create more revenue in the same business operation cycle. Green practice activities will cost a great deal in technological innovation, production, and other aspects. Yet, there is a large uncertainty in green benefits, and most companies need help achieving their green goals [39]. Thirdly, the value-added service revenue is set. The package of service demands is composed of several value demands, i.e., some are core value demands and additional value demands. The greening of manufacturing enterprises requires a large amount of capital investment upfront, and the resulting huge sunk cost, other value-added services can share the cost.

In summary, manufacturing companies obtain better performance through profitability models, and manufacturing companies with good green value creation can effectively contribute to profitability models through revenue model innovation, cost structure adjustment, capital efficiency improvement, and increased financial support, thus achieving improved corporate performance. This study proposes the following hypotheses.

H2: The profit model mediates between green value creation and corporate performance.

H2a: The profit model significantly and positively affects environmental performance.

H2b: The profit model significantly and positively affects economic performance.

H2c: The profit model significantly and positively affects customer value.

### 2.3 Regulation of green dynamic capacity role

(1) The moderating role of green dynamic capabilities in the relationship between green value creation and corporate performance

The uncertainty of the external environment requires companies to have the dynamic ability to integrate and allocate organizational resources effectively. Due to the increasing environmental degradation, more and more companies are aware of the importance of environmental protection. For this reason, Li (2013) [40], Li (2018) [41], Xing and Yu (2020) proposed that dynamic capabilities should be integrated into green elements and put forward the concept of green dynamic capabilities [42]. Chen (2013) considered green dynamic capabilities as the ability of enterprises to use existing resources to update and develop green organizational capabilities and respond to dynamic market changes, which catalyzes enterprises’ green transformation [43].

Companies need to generate products and services that meet green needs through each value-creating activity in the value network to provide to customers and transfer activities that reduce energy consumption and environmental pollution to manufacturing companies to form cost reductions. These activities promote the improvement of environmental performance on the one hand and improve economic performance on the other. When the environmental payoff can be translated into improving economic performance, the external economics of environmental protection is transformed into the internal motivation mechanism of the enterprise. Sheng and Ge’s (2019) research and development found that green consumption requires extra efforts from consumers, such as paying environmental premiums for green products and shifting old consumption habits, and requires consumers to make decisions about perceived ecological benefits and personal gains and losses [44]. Only under the green dynamic capability do manufacturing companies clarify the interests of customers and stakeholders and, on this basis, develop the content of the transaction of green products or services, the transaction mode, and complete the value acquisition. Such as accessible mode, attract customers lock around the green products to provide related products or services and then obtain stable income. With the continuous improvement of the digital capability of enterprises, intelligent interconnection of green products, products as a carrier to deliver green services and bring sustainable profit for enterprises. The industrial Internet platform is free for customers on one side and charges for customers or third parties on the other. Cross-subsidization allows the free charging approach to expand the customer base and thus gain economies of scale.

Scholars have verified the positive impact of green dynamic capabilities on green innovation [45], environmental innovation [38], competitive advantage [46], and firm performance [47]. Therefore, this paper argues that green dynamic capabilities play an important role in influencing green value creation and profit model, and only when companies have specific green dynamic capabilities manufacturing companies set regulations, revenue models, and cost structures to target users and the results of green value creation can be effectively shared to reach the desired goals. Stakeholders can better carry out green value creation activities.

In summary, the following hypothesis is proposed in this paper.

H3: Green dynamic capabilities play a positive moderating role between green value creation and corporate performance.

H3a: Green dynamic capabilities play a positive moderating role between green value creation and environmental performance.

H3b: Green dynamic capabilities play a positive moderating role between green value creation and economic performance.

H3c: Green dynamic capability plays a positive moderating role between green value creation and customer value.

(2) Moderating role of green dynamic capability between green value creation and profit model

Zhou (2017) suggested that companies must develop a value proposition that meets customers’ willingness to demand and use it to build a sustainable and profitable business model that provides customers with goods or services at an acceptable price and quality. Customers’ attitudes toward products affect their acceptance of green products or services, willingness to pay, etc. Companies enhance their market power and set price mechanisms by targeting customers. Especially in platform companies, customer lock-in can avoid or reduce value slippage [48].

Existing studies have focused on the match between green dynamic capabilities using digital technologies that enable green value creation and profitability models. Hou et al. (2014) pointed out that the core of digital technology is prediction and personalization, and companies can precisely target customers’ willingness to pay for their needs through the analysis and mining of massive data. Promote the gradual change of profit sources from fee-based models such as sale (rental) and licensing to free and cross-border fee models [49]. Accordingly, attract customers, expand the size of the customer base, target customers, and build new profit centers. Davenport (2014) stated that data analysis helps companies optimize their pricing system, reduce corporate costs, and improve their profit margins [50]. Big data identifies customers’ segmentation needs, enables precise marketing, identifies customers’ willingness to pay, optimizes the cost structure, and forms financial payment methods, such as Jingdong White Strip, Jingdong Finance, and others. Expand the revenue of enterprises and open up new profit models. Manufacturing enterprises can analyze the information related to the constraints and incentives of government environmental regulations that customers are subject to in the production and sales process based on their massive data, tap into the pain points of customer payments, and design innovative solutions for for-profit models through intelligent analysis. Such as lowering the fees for manufacturing companies to provide green products and raising the fees for supporting green services or solutions, influencing customer preferences, increasing interaction and attachment with customers, and thus improving business performance. The application of blockchain technology changes the transaction method for value acquisition. Zhang and Zhu (2020) Blockchain technology processes credit system reconstruction, removes the centralized intermediary guarantee process, and makes online payment possible peer-to-peer, thus realizing secure value delivery over long distances on an open platform. Learning that exchange value can be done directly between peer-to-peer reduces transaction costs, changes the way exchange value is realized, and brings value-added effects. It also provides new ideas for green value acquisition, and implementing this concept requires the development of the role of green dynamic capabilities [51]. Cui et al. (2021) Green dynamic capabilities promote the establishment and adjustment of profitability models as well as respond to changes in customer payment needs, goods, services, and other demands in green markets by acquiring and integrating information related to customer demand, willingness to pay, market demand, and business development [52].

Therefore, when the green dynamic capability of manufacturing enterprises is high, the contribution of green value creation to the profit model is stronger. When the green dynamic capability of manufacturing enterprises is at a high level, enterprises can more deeply explore the customers’ willingness to need green products or green services, expected utility, and willingness to pay, and innovate the profit model accordingly so that the profit model is more in line with the customers’ willingness to need. The more the customers rely on the enterprise’s green products or services, the stronger the isolation mechanism of the enterprise. A stable customer source is the premise and foundation for enterprises to obtain economic benefits. Conversely, when the enterprise’s green dynamic capability is weak, the enterprise either pays less attention to obtaining information related to the customer’s willingness to pay or the enterprise has difficulty in obtaining relevant information, and the green value created by the enterprise is difficult to match with the profit model. The motivation and enthusiasm of customers to express their ideas and opinions about products or services to the enterprise will be reduced, weakening the enterprise’s lock on customers. It is challenging to establish segregation mechanisms to obtain a stable revenue stream. That is, green dynamic capability plays a positive moderating role between green value creation and profitability models.

H4: Green dynamic capabilities play a positive moderating role between green value creation and profitability models.

In summary, the theoretical assumptions and the theoretical model of this study can be derived from the above analysis, and the relevant values of this study are summarized in Table 1 below.

**Table 1:** Summary of research hypotheses related to green value capture in manufacturing firms.

Assumptions	Assume the specific content
H1	Green value creation significantly and positively affects corporate performance
H1a	Green value creation significantly and positively affects environmental performance
H1b	Green value creation significantly and positively affects economic performance
H1c	Green value creation significantly and positively affects customer value
H2	The profit model mediates between green value creation and corporate performance
H2a	The profit model significantly and positively affects environmental performance
H2b	The profit model significantly and positively affects economic performance
H2c	The profit model significantly and positively affects customer value
H3	Green dynamic capabilities play a positive moderating role between green value creation and corporate performance
H3a	Green dynamic capabilities play a positive moderating role between green value creation and environmental performance
H3b	Green dynamic capabilities play a positive moderating role between green value creation and economic performance
H3c	Green dynamic capability plays a positive moderating role between green value creation and customer value
H4	Green dynamic capabilities play a positive moderating role between green value creation and profitability models

## 3 Methodology

### 3.1 Green value acquisition mechanism model construction

**Fig 1:**
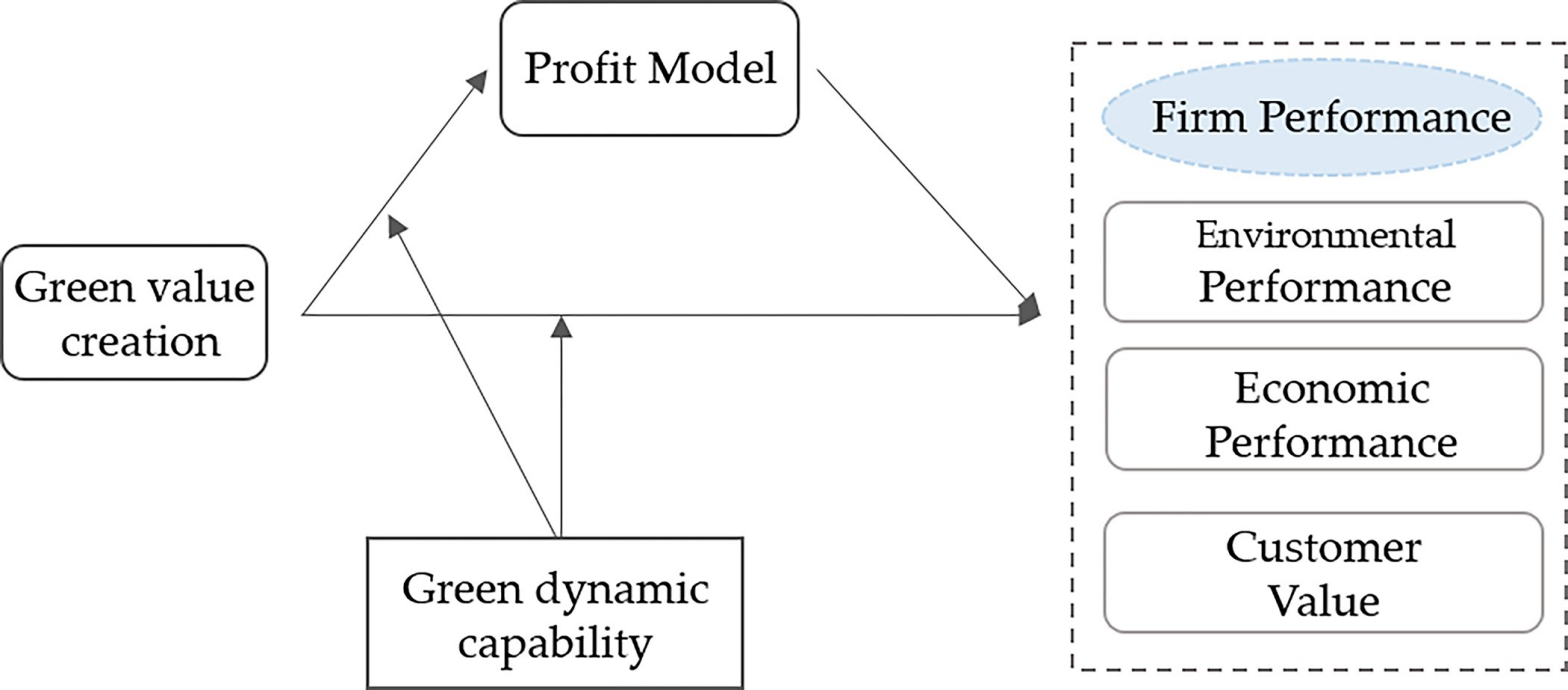
Model of green value acquisition mechanism of manufacturing companies.

Through the above analysis of the impact of green value creation, profitability model, and green dynamic capability of manufacturing enterprises on enterprise performance, as well as the relevant theoretical hypotheses proposed, a hypothetical model of green value acquisition mechanism of manufacturing enterprises can be summarized, as shown in Fig 1.

### 3.2 Scale design

The dependent, independent, moderating, and control variables covered in this chapter are all measured by drawing on a 5-point Likert scale, from 1 to 5, representing very weak, weak, moderate level, strong, and very strong, respectively, except for individual control variables that require special description.

(1) Green value creation. Referring to the study of Song et al. (2018) proposed a scale for designing value-creation activities based on the involvement of major partners in four aspects: product design, operational processes, product development, and strategic planning [53]. The study by Xin and Li (2022) proposed the scale based on the four aspects of new product design and development, operational processes, and strategic planning optimization that companies can accomplish in collaboration with their partners [54]. In addition, this study also revised and added to the questionnaire based on multiple rounds of revisions by experts, and the specific questions are shown in Table 2.

**Table 2:** Measurement scale for green value creation.

Variables	Number	Title
Green Value Creation	GVP1	Customer participation in the whole process of green product and service design and development
	GVP2	Key partners are involved in the entire process of designing and developing green products and services
	GVP3	Key partner engagement to improve operational processes
	GVP4	Enterprises and partners establish risk assessment and avoidance mechanisms
	GVP5	Companies and partners provide green solutions to members of the entire value network

(2) Profitability model Referring to the study of Wang et al. (2015), the profitability model scale was proposed to be designed based on three elements: revenue source, cost structure, and asset utilization [36]. Chang et al. (2017) study suggested a design of the value realization scale based on three aspects: the quantity and stability of revenue sources, cost control advantages, and convenient payment methods [55]. Qian et al. (2018) constructed value acquisition from two dimensions: revenue pattern and cost structure [56]. Li et al. (2019) took manufacturing servitization as the research object. They proposed the measurement dimensions of the manufacturing servitization profitability model in terms of cost structure, revenue model, and pricing strategy [38]. Zhang et al. (2022) conducted a review study of business model research in the context of digitalization. They proposed the combination of digital and technology to highlight the traditional profitability model and broaden access to corporate value through platforms and free models [37]. Dun and Chen (2019) proposed that the diversified profit models of enterprises in the sharing economy diversify the risks of enterprises while increasing their benefits. The greening process of manufacturing enterprises requires a long period of investment and a huge amount of investment, superimposed on the uncertainty of the market; the difficulty of financial financing has a great impact on the greening process of enterprises, and for this reason, the profit model incorporates financial support into the scope of analysis [57]. Combining the existing scales and the results of interviews and expert discussions, the scale of profitability model was designed in terms of cost control, revenue model, asset utilization efficiency, and financial support. In addition, this study also revised and supplemented the questionnaire based on multiple rounds of revisions by experts, and the specific questions are shown in Table 3.

**Table 3:** Measurement scale of profitability model.

Variables	Number	Title
Profit Model	PM1	Companies are able to control the input costs of major factors of production at a lower level in the industry or significantly reduce or remove cost items that are prevalent in the industry
	PM2	The company forms a revenue pyramid, and there are many types of customers who pay, and the revenue is stable.
	PM3	Companies consolidate and utilize external assets and have fast overall asset turnover
	PM4	Companies get more financial support through green behavior

(3) Corporate Performance. Manufacturing enterprise performance includes three dimensions: environmental performance, economic performance, and customer value obtained from value creation.

Environmental performance. The scale of the environmental performance of manufacturing enterprises mainly refers to [58, 59] Liu (2020) and Meng (2022) [60] related studies, which measure pollution emissions and environmental accidents occurring in manufacturing enterprises and combined with the actual situation of this paper, the content of the improved scale includes four questions, respectively, from the reduction of three wastes in manufacturing enterprises, the use of hazardous and toxic materials, the frequency of environmental accidents and the environmental improvement of enterprises The scale includes four items, which measure the reduction of three wastes, the use of hazardous and toxic materials, the frequency of environmental accidents, and the improvement of the environment.

Economic performance. The scale of the economic performance of manufacturing enterprises mainly refers to the relevant study by Liu (2020) [61], which measures the cost reduction and revenue rise of manufacturing enterprises. Combined with this paper, the content of the improved scale includes five questions, which are measured in terms of the recycling of materials, energy consumption, three waste emission costs, treatment costs, and the contribution of green products and services to the corporate profits of manufacturing enterprises. The scale of customer value mainly refers to the relevant research of Di (2015) [62] and includes three questions. It measures customer engagement, satisfaction, and loyalty, respectively. The specific questions of the above three dimensions are shown in Table 4.

**Table 4:** Measurement scale for green value capture.

Variables	Number	Title
Environmental Performance	EP1	In the last three years, the enterprise three waste reduction
	EP2	Reduction in the use of hazardous/toxic and harmful materials by enterprises in the last three years
	EP3	Reduction in the frequency of environmental accidents in enterprises in the last three years
	EP4	In the last three years, the environmental situation of enterprises has improved
Economic Performance	ENP1	In the last three years, the company reused and recycled materials to reduce procurement costs
	ENP2	Reduction in the cost of energy consumption of the Company in the last three years
	ENP3	Over the last three years, the Company has seen a reduction in waste disposal costs
	ENP4	In the last three years, the enterprise’s waste discharge costs have decreased
	ENP5	In the past three years, the green products and services provided by our company have greatly increased our profits.
Customer Value	CV1	In the last three years, business users have participated in the whole process of green products and services of this enterprise
	CV2	In the last three years, business users’ satisfaction with green products and services has increased significantly
	CV3	In the last three years, the company’s old users have recommended our green products and services to more new customers

(4) Moderating variables

Xing and Yu (2020) [42] proposed that green dynamic capability should be divided into two dimensions: green resource acquisition capability and green resource integration capability. The scale of green dynamic capability mainly refers to the relevant studies of Huang et al. (2015) [63] and Cao and Zhao (2017) [46], which measure the green environmental adaptation ability, green resource integration ability and organizational absorption ability of enterprises. Combined with the actual paper, the content of the improved scale contains a total of five questions, which are measured in three dimensions: perceived opportunity ability, integration and utilization ability, and reconfiguration and transformation ability. Measurement scale of green dynamic capacity can be shown in Table 5.

**Table 5:** Measurement scale of green dynamic capacity.

Variables	Number	Title
Green Dynamic Capabilities	GDC1	Companies can obtain timely information about changes in customer green needs through various channels
	GDC2	Companies can quickly identify and recognize market opportunities or potential threats arising from government environmental policies
	GDC3	The implementation of green strategies by enterprises has enabled them to access a large number of external resources
	GDC4	Companies use integrated internal and external resources to improve efficiency and effectiveness
	GDC5	Companies design green management mechanisms, deploy and integrate resources and capabilities

### 3.3 Data collection

This study collected research data using a questionnaire survey launched by manufacturing companies in Hebei Province, Heilongjiang Province, and the Yangtze River Delta region. The survey was focused on the period of November 2021-February 2022. To avoid the effect of homophily bias, this study used a two-stage release of the questionnaire. In the first stage (2 November 2021 to—5 December 2021), the researcher released the questionnaires to the R&D managers, technical directors, and other executives of manufacturing companies to obtain demographic information, corporate information, and evaluation information of green value and green dynamic capabilities. After several instructions, 320 questionnaires were finally received, and 308 valid questionnaires were obtained after excluding those containing missing values. In the second stage (1 January 2022 to 3 February 2022), the researcher released questionnaires to financial managers, marketing managers, and other executives from the 308 questionnaire companies according to the enterprise number to obtain the evaluation information of the profitability model of the manufacturing companies and the performance of the companies, and finally, 280 questionnaires were received. After excluding the questionnaires with too short answer time, incomplete answer information, and regular answers and matching the data of the two phases according to the enterprise number, 263 valid questionnaires were finally obtained. The overall effective recovery rate of the questionnaires was 82.2%. Overall, the effective recovery rate of questionnaires is high.

#### (1) Descriptive statistical analysis

In this study, descriptive statistical analysis was conducted on the 263 validly recovered samples, and the results of the analysis are shown in Table 6. As seen in Table 6, the subjects who participated in this study had the following characteristics.

**Table 6:** Results of descriptive statistical analysis of the sample.

Variables	Classification	Percentage
Age	Under 29 years old	33.8%
	30–40 years old	38.0%
	40–50 years old	15.2%
	Over 50 years old	12.9%
Gender	Male	53.2%
	Female	46.8%
Business Age	≤10 years	31.6%
	10–15 years	32.3%
	15–20 years	12.9%
	≥20 years	23.2%
Enterprise size	≤2000 people	37.60%
	2000–10,000 people	25.10%
	10,000–50,000 people	23.60%
	50,000–100,000 people	5.30%
	More than 100,000 people	8.40%
Nature of business	Wholly State-Owned	39.9%
	Private Enterprises	48.7%
	Wholly Foreign Owned	3.8%
	Joint Ventures	7.6%
Industry of the company	Metal manufacturing	22.4%
	Electronic technology manufacturing	23.2%
	Light textile industry	18.3%
	Mechanical industry	16.7%
	Chemical industry	19.4%

As far as the respondents are concerned, their age group is concentrated the most: 30–40 years old, accounting for 38.0%, followed by those under 29 years old, accounting for 33.8%; and the least number of subjects in the age group above 50 years old, accounting for 12.9%. Regarding gender, 53.2% of the subjects were male, and 46.8% were female, with a relatively moderate ratio of male to female.

As far as the researched enterprises are concerned, those with 10–15 years of age are the most numerous, accounting for 32.3%; those with less than ten years of age are the next oldest, accounting for 31.6%; those with 15–20 years of age are the least, accounting for 12.9%. In terms of enterprise scale, enterprises with less than 2,000 employees accounted for 37.6%; enterprises with 2,000–10,000 employees accounted for 25.1%; enterprises with 10,000–50,000 employees accounted for 23.6%; enterprises with 50,000–100,000 employees accounted for 5.3%; enterprises with more than 100,000 employees accounted for 8.4%. Regarding the nature of enterprises, 39.9% of wholly state-owned enterprises, 48.7% of private enterprises, 3.8% of wholly foreign-owned enterprises, and 7.6% of joint ventures. In terms of the industries to which the enterprises belong, electronic technology manufacturing enterprises account for the highest proportion of 23.2 percent, followed by metal manufacturing enterprises with a proportion of 22.4 percent, light textile industry enterprises with a proportion of 18.3 percent, machinery manufacturing enterprises with a proportion of 16.7 percent, and chemical enterprises with a proportion of 19.4 percent. Overall, the sample selected for this study is relatively balanced and representative.

Descriptive statistical analysis was also conducted for the variables involved in this study, and the means and standard deviations of each variable are shown in Table 7. As can be seen from Table 7, the mean values of the variables green value creation, profitability model, green dynamic capability, corporate environmental performance, corporate economic performance, corporate customer value, and corporate performance are greater than the median value of 3, indicating that the mean values of the variables involved in this study are above the score of 2.5.

**Table 7:** Results of descriptive statistical analysis of variables.

	Average value	Standard deviation
Green Value Creation	3.78	1.11
Profit Model	3.77	1.21
Green Dynamic Capability	4.07	0.80
Corporate Environmental Performance	3.59	1.12
Corporate Economic Performance	3.66	1.09
Corporate Customer Value	3.55	1.19
Corporate Performance	3.61	0.96

#### (2) Common method deviation test

Standard method bias refers to an artificial covariation between explanatory and explanatory variables caused by the singularity of the research data source, the influence of the research environment, and the semantic ambiguity of the research questions. This can have an impact on the reliability and accuracy of the research findings. The main variables in this study were measured in a self-reported manner by the subjects, and the relationships between the variables are inevitably affected by common method bias, so it is essential to conduct common method bias tests in this study. In the common method test, Harman’s single factor test was used in this paper, and all measured topics of green value creation, profitability model, green dynamic capability, corporate environmental performance, corporate economic performance, and corporate customer value were simultaneously included in the exploratory factor analysis structure for unrotated principal component analysis, and the results are shown in Table 8, from which it can be seen that a total of six factors were extracted, and the cumulative total explained variance was 79.671% and the first factor explained 44.717% of the total variance, which did not exceed the critical value of 50%, thus showing that there was no serious common method bias in this study.

**Table 8:** Results of unrotated principal component analysis.

Variables	Initial Eigenvalue			Extraction of the sum of squares of loads		
	Total	Variance %	Cumulative %	Total	Variance %	Cumulative %
1	11.626	44.717	44.717	11.626	44.717	44.717
2	3.198	12.301	57.018	3.198	12.301	57.018
3	1.957	7.528	64.546	1.957	7.528	64.546
4	1.710	6.576	71.122	1.710	6.576	71.122
5	1.211	4.658	75.780	1.211	4.658	75.780
6	1.012	3.891	79.671	1.012	3.891	79.671

### 3.4 Reliability test

#### (1) Reliability test

Reliability is a measure of a scale’s reliability, stability, and consistency. The larger the reliability value of a scale, the better the stability and reliability of the scale. In the existing studies, most scholars use the internal consistency coefficient, Cronbach’s α, to test the reliability of the scale. Generally, Cronbach’s α value greater than 0.7 indicates the scale has good reliability. According to the study of Abboh et al. (2022) [64], Cronbach’s alpha in Table 9 meets the requirements of further empirical research.

**Table 9:** Cronbach’s alpha coefficients for the variables.

Variables	Cronbach’s Alpha	Number of items
Green Value Creation	0.929	5
Profit Model	0.928	4
Green Dynamic Capability	0.904	5
Environmental Performance	0.934	4
Economic Performance	0.921	5
Customer Value	0.917	3
Corporate Performance	0.937	12

The Cronbach’s α values for each scale in this study are shown in Table 9. As seen in Table 9, the Cronbach’s alpha coefficient for green value creation is 0.929, the Cronbach’s alpha coefficient for profitability model is 0.928, the Cronbach’s alpha coefficient value for green dynamic capability is 0.904, the Cronbach’s alpha coefficient value for corporate environmental performance Cronbach’s alpha coefficient value is 0.934, Cronbach’s alpha coefficient for firm economic performance is 0.921, Cronbach’s alpha coefficient value for strong customer value is 0.917, and Cronbach’s alpha coefficient values for firm performance’s α value is 0.937, respectively. The Cronbach’s α values of each variable are greater than 0.7, indicating that the scale used in this study has high reliability and good reliability tests.

#### (2) Validity test

The validity test is a test of the validity of a scale to reflect the degree of agreement between the findings and what is wanted to be examined. This study focuses on testing the convergent validity of the scale.

Convergent validity is the degree to which a variable is better measured by its indicators. It is generally judged by standardized factor loadings, average variance extracted (AVE), and combined reliability (CR). The higher the standardized factor loadings, the better the indicator is explained by the variable, and the better the indicator reflects the characteristics of the variable; the higher the AVE value, the better the potential variable is able to explain its corresponding question. The standardized factor loadings for each item of the scale are greater than 0.5, AVE is greater than 0.5, and CR is greater than 0.7, which means that the scale has good convergent validity. According to the study of Abboh et al. (2022) [64], composite reliability and AVE values in Table 10 meet the requirements of further empirical research.

**Table 10:** Convergent validity test results.

Variables	Title	Factor load	AVE	CR
Green Value Creation	CGV1	0.874	0.728	0.930
	CGV2	0.784		
	CGV3	0.837		
	CGV4	0.862		
	CGV5	0.905		
Profit Model	PM1	0.935	0.766	0.929
	PM2	0.862		
	PM3	0.846		
	PM4	0.856		
Green Dynamic Capability	GDC1	0.839	0.656	0.905
	GDC2	0.825		
	GDC3	0.845		
	GDC4	0.843		
	GDC5	0.687		
Corporate Environmental Performance	EP1	0.945	0.787	0.936
	EP2	0.835		
	EP3	0.857		
	EP4	0.907		
Corporate Economic Performance	ENP1	0.947	0.709	0.924
	ENP2	0.792		
	ENP3	0.802		
	ENP4	0.798		
	ENP5	0.862		
Corporate Customer Value	CV1	0.925	0.788	0.918
	CV2	0.856		
	CV3	0.881		

In this study, the standardized factor loadings, AVEs, and CRs of each question in the Green Value Creation, Profitability Model, Green Dynamic Capability, Corporate Environmental Performance, Corporate Economic Performance, and Corporate Customer Value scales were calculated using AMOS 24.0 software, and the results are shown in Table 10. The average variance extracted (AVE) of each variable is greater than 0.5, and the combined reliability (CR) of each variable is greater than 0.7, which indicates that the scale used in this study has good convergent validity.

## 4 Empirical analysis

### 4.1 Correlation analysis of variables

In order to clarify the relationship between the variables involved in this study, the Pearson correlation coefficient method was used to measure the correlation between the variables in this study, and the results are shown in Table 11. From Table 11, it can be seen that.

1. Corporate green value creation was significantly and positively correlated with corporate environmental performance (r = 0.459, p<0.01), corporate economic performance (r = 0.530, p<0.01) and corporate customer value (r = 0.574, p<0.01).
2. Corporate green value creation was significantly and positively correlated with profitability model (r = 0.552, p<0.01).
3. Corporate profitability model is significantly and positively related to corporate environmental performance (r = 0.492, p<0.01), corporate economic performance (r = 0.641, p<0.01) and corporate customer value (r = 0.620, p<0.01).

**Table 11:** Correlation analysis of variables.

	1	2	3	4	5	6	7	8	9
1	1								
2	0.227**	1							
3	-0.031	0.095	1						
4	0.089	-0.062	0.045	1					
5	0.031	-0.040	-0.162**	0.060	1				
6	-0.044	-0.141*	-0.213**	-0.08	0.552**	1			
7	-0.044	0.017	-0.042	-0.017	0.323**	0.356**	1		
8	0.000	-0.139*	-0.160**	0.021	0.459**	0.492**	0.235**	1	
9	-0.005	-0.086	-0.281**	-0.017	0.530**	0.641**	0.192**	0.568**	1
10	0.046	-0.136*	-0.143*	0.015	0.574**	0.620**	0.325**	0.576**	0.623**

Note: N = 263

* indicates P<0.05

** indicates P<0.01

*** indicates P<0.001

The correlation analysis among the variables initially verified the hypotheses proposed in this study and provided a preliminary basis for testing the hypotheses in the subsequent study.

In Table 1, 1 represents the age of the enterprise, 2 represents the size of the enterprise, 3 represents the nature of the enterprise, 4 represents the industry of the enterprise, 5 represents green value creation, 6 represents the profit model, 7 represents green dynamic capability, 8 represents enterprise environmental performance, 9 represents enterprise economic performance, and 10 represents enterprise customer value.

### 4.2 Main effects test

In this study, the main effect of green value creation on firm performance was tested by multivariate hierarchical regression analysis in SPSS 26.0 software, and the results are shown in Table 12. Model 2 in Table 12 shows that green value creation has a significant positive effect on firm performance (β = 0.583, p<0.001), which proves that hypothesis H1 is valid. As seen from model 4, green value creation has a significant positive impact on environmental performance (β = 0.442, p<0.001), which proves that hypothesis H1a holds. As seen in model 6, green value creation has a significant positive impact on economic performance (β = 0.500, p<0.001), which proves that hypothesis H1b is valid. As seen from model 8, green value creation has a significant positive impact on customer value (β = 0.563, p<0.001), which proves that hypothesis H1c is valid.

**Table 12:** Results of regression analysis of green value creation on corporate performance.

Variables	Corporate Performance		Environmental Performance		Economic Performance		Customer Value	
	Model 1	Model 2	Model 3	Model 4	Model 5	Model 6	Model 7	Model 8
Business Age	0.032	0.017	0.023	0.011	0.001	-0.012	0.073	0.058
Enterprise size	-0.122	-0.107*	-0.129*	-0.117***	-0.061	-0.048	-0.140*	-0.125*
Nature of business	-0.227***	-0.133**	-0.148***	-0.077	-0.274***	-0.193***	-0.128*	-0.037
Industry of the company	0.005	-0.032	0.018	-0.010	-0.009	-0.041	0.006	-0.030
Green Value Creation		0.583***		0.442***		0.500***		0.563***
R2	0.071	0.400	0.042	0.232	0.068	0.311	0.041	0.348
F-value	4.961**	34.275***	2.832*	15.490***	5.793***	24.664***	2.734*	27.389***

Note: N = 263

* indicates P<0.05

** indicates P<0.01

*** indicates P<0.001

### 4.3 Testing the mediating effect of the profitability model

This study proposes the hypothesis that the profitability model mediates between green value creation and corporate performance (H2) and the hypothesis that the profitability model mediates between green value creation and three sub-dimensions of environmental performance, economic performance, and customer value (H2a, H2b, and H2c). In this study, the stepwise regression method and Bootstrap method will be used to test the mediating effect of the profitability model.

#### (1) Test of the role of green value creation on profitability model

The study used the stepwise regression method to verify the effect of green value creation on the profitability model, and the test results are shown in Table 13; as shown by model 10 in Table 13, green value creation has a significant positive effect on the profitability model (β = 0.537, p<0.001).

**Table 13:** Results of regression analysis of green value creation on profitability model.

Variable Name	Profit Model	
	Model 9	Model 10
Company Age	-0.015	-0.029
Enterprise size	-0.123*	-0.109*
Nature of business	-0.198**	-0.111*
Industry of the company	-0.077	-0.111*
Green Value Creation		0.537***
R2	0.066	0.346
F-value	4.578**	27.159***

Note: N = 263

* indicates P<0.05

** indicates P<0.01

*** indicates P<0.001

#### (2) Test of the role of the profitability model on corporate performance

To verify the effect of the profitability model on firm performance, the study still uses the stepwise regression method to test the results, as shown in Table 14; as seen from model 12 in Table 14, the profitability model has a significant positive effect on firm performance (β = 0.667, p<0.001). As seen from model 14, the profitability model significantly positively affects corporate environmental performance (β = 0.477, p<0.001). As seen by model 16, the profitability model has a significant positive effect on the firm’s economic performance (β = 0.614, p<0.001). As seen by model 18, the profitability model significantly positively affects corporate customer value (β = 0.618, p<0.001).

**Table 14:** Results of regression analysis of profitability model on firm performance.

Variable Name	Corporate Performance		Environmental Performance		Economic Performance		Customer Value	
	Model 11	Model 12	Model 13	Model 14	Model 15	Model 16	Model 17	Model 18
Business Age	0.032	0.042	0.023	0.030	0.001	0.011	0.073	0.082
Enterprise size	-0.122	-0.040	-0.129*	-0.07	-0.061	0.015	-0.140*	-0.063
Nature of business	-0.227***	-0.095*	-0.148*	-0.054	-0.274***	-0.153**	-0.128*	-0.006
Industry of the company	0.005	0.056	0.018	0.055	-0.009	0.039	0.006	0.053
Profit Model		0.667***		0.477***		0.614***		0.618***
R2	0.071	0.487	0.042	0.254	0.082	0.434	0.041	0.397
F-value	4.961**	48.704***	2.832*	17.507***	5.793***	39.443***	2.734*	33.889***

Note: N = 263

* indicates P<0.05

** indicates P<0.01

*** indicates P<0.001

#### (3) Testing the mediating role of profitability models in the relationship between green value creation and corporate performance

To test the mediating role of the earnings model, the study included the control variables, green value creation, and earnings model simultaneously in the regression equation and constructed a regression of the mediating effect of the earnings model; the results of the regression analysis are shown in Table 15, as seen in 6.15, the regression coefficient of green value creation on firm performance decreased from 0.583 in Model 2 of Table 12 to 0.320 in Model 19 of Table 15 (p< 0.001), indicating that profitability model plays a partially mediating role between green value creation and corporate performance, and therefore hypothesis H2 holds. The regression coefficient of green value creation on environmental performance decreases from 0.442 in Model 4 of Table 12 to 0.266 in Model 20 of Table 15 (p< 0.001), indicating that the profitability model plays a partially mediating role between green value creation and environmental performance. Thus, hypothesis H2a holds. The regression coefficient of green value creation on economic performance decreases from 0.500 in Model 6 of Table 12 to 0.243 in Model 21 of Table 15 (p<0.001), indicating that the profitability model plays a partially mediating role between green value creation and economic performance. Therefore, hypothesis H2b is valid. Similarly, the regression coefficient of green value creation on customer value decreases from 0.563 in Table 12 Model 8 to 0.330 in Table 15 Model 22 (p<0.001), indicating that the profitability model plays a partially mediating role between green value creation and customer value. Therefore, hypothesis H2c is valid.

**Table 15:** Results of the regression analysis of the mediating effect of the profit model.

Variable Name	Corporate Performance	Environmental Performance	Economic Performance	Customer Value
	Model 19	Model 20	Model 21	Model 22
Company Age	0.031	0.021	0.002	0.071
Enterprise size	-0.054	-0.081	0.004	-0.077
Nature of business	-0.078	-0.040	-0.140**	0.011
Industry of the company	0.022	0.026	0.013	0.018
Green Value Creation	0.320***	0.266***	0.243***	0.330***
Profit Model	0.488***	0.328***	0.479***	0.434***
R2	0.556	0.302	0.474	0.471
F-value	53.453***	18.568***	38.470***	38.002***

Note: N = 263

* indicates P<0.05

** indicates P<0.01

*** indicates P<0.001

The study further tested the mediating effect of the earnings model using the Bootstrap method, and the results are presented in Table 16. As seen in Table 16, the mediating effect of the earnings model between green value creation and firm performance is significant (Effect = 0.228, SE = 0.038, 95% CI = [0.156,0.304] and does not contain 0, further proving that hypothesis H2 holds. The significant mediating effect of the profitability model between green value creation and environmental performance (Effect = 0.178, SE = 0.043,95% CI = [0.101,0.268], excluding 0, further proves that hypothesis H2a is valid. The significant mediating effect of the profitability model between green value creation and economic performance (Effect = 0.253, SE = 0.042,95%CI = [0.173,0.339], excluding 0, further proves that hypothesis H2b holds. Similarly, the mediating effect of the profitability model between green value creation and customer value is significant (Effect = 0.251, SE = 0.051, 95% CI = [0.163,0.360], excluding 0, further proving that hypothesis H2c is valid.

**Table 16:** Results of bootstrap mediated effects analysis.

Paths	Effect	BootSE	BootLLCI	BootULCI
Green Value Creation → Profit Model → Corporate Performance	0.228	0.038	0.156	0.304
Green Value Creation → Profit Model → Environmental Performance	0.178	0.043	0.101	0.268
Green Value Creation → Profit Model → Economic Performance	0.253	0.042	0.173	0.339
Green Value Creation → Profit Model → Customer Value	0.251	0.051	0.163	0.360

### 4.4 Regulation effect test

#### (1) Examination of the moderating effect of green dynamic capabilities on the relationship between green value creation and corporate performance

The study hypothesized that green dynamic capability has a positive moderating effect between green value creation and firm performance (H3), and this study used model 1 in Process to test the moderating effect and the test results are shown in Table 17.

**Table 17:** Test of the moderating effect of green dynamic capabilities between green value creation and firm performance.

Variables	Corporate Performance	
	Coeff	95% CI
Constants	4.055***	[3.726,4.384]
Business Age	-0.010	[-0.089,0.069]
Enterprise size	-0.083*	[-0.155,-0.01]
Nature of business	-0.170**	[-0.275,-0.064]
Industry of the company	-0.006	[-0.067,0.055]
Green Value Creation	0.455***	[0.372,0.539]
Green Dynamic Capability	0.105	[-0.009,0.219]
Green Value Creation* Green Dynamic Capability	0.284***	[0.184,0.384]
R2	0.474	
F	32.846***	

Note: N = 263

* indicates P<0.05

** indicates P<0.01

*** indicates P<0.001

As seen in Table 17, in the model with the interaction term of green value creation, green dynamic capability, green value creation and green dynamic capability as independent variables and firm performance as dependent variable, the coefficient of the interaction term of green value creation and green dynamic capability is positive with a 95% confidence interval of [0.184,0.384] not containing 0, thus indicating that the interaction term of green value creation and green dynamic capability has a This indicates that the interaction term between green value creation and green dynamic capabilities has a significant positive effect on firm performance (β = 0.284, p<0.001). In addition, green value creation also has a significant positive effect on firm performance (β = 0.455, p<0.001), tentatively indicating a positive moderating effect of green dynamic capabilities between green value creation and firm performance, and hypothesis H3 is supported.

**Fig 2:**
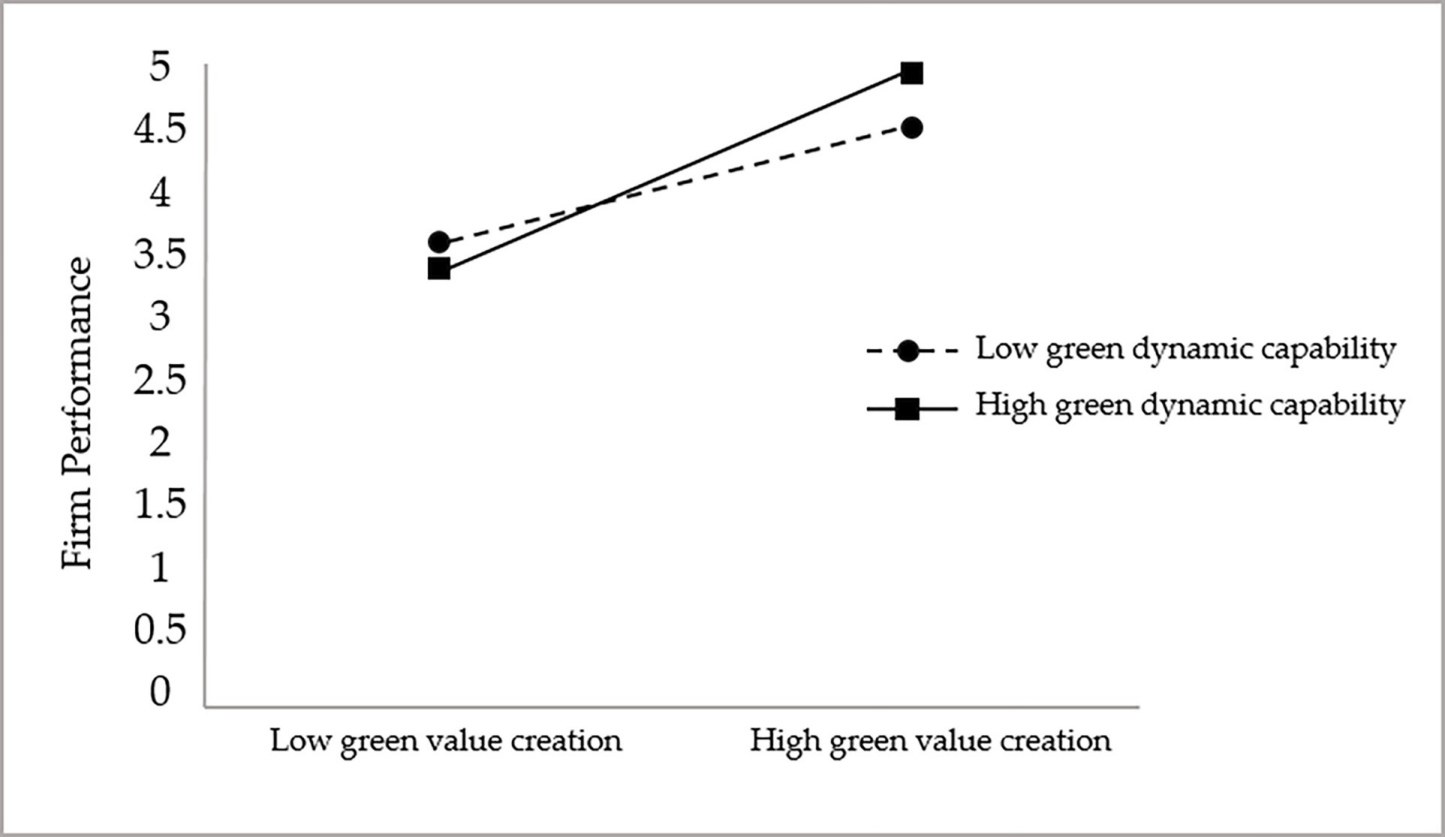
The moderating effect of green dynamic capability on the relationship between green value creation and corporate performance.

To more visually and clearly show the moderating effect of green dynamic capabilities in the relationship between green value creation and firm performance and to further test hypothesis H3, this study plots the moderating effect, as shown in Fig 2.

As seen in Fig 2, the slope under high green dynamic capability is greater than that under low green dynamic capability, thus indicating that the positive relationship between green value creation and firm performance is stronger under high green dynamic capability compared to low green dynamic capability, and hypothesis H3 is further supported.

#### (2) Test of the moderating effect of green dynamic capabilities on the relationship between green value creation and each dimension of corporate performance

The study hypothesized that green dynamic capability has a positive moderating effect between green value creation and corporate environmental performance (H3a), green dynamic capability has a positive moderating effect between green value creation and corporate economic performance (H3b), and green dynamic capability has a positive moderating effect between green value creation and customer value (H3c), to verify the moderating effect of green dynamic capability in the relationship between green value creation and To verify the moderating effect of green dynamic capability in the relationship between green value creation and each dimension of enterprise performance, this study also adopts model 1 in Process to test the moderating effect, and the test results are shown in Table 18.

**Table 18:** Test of moderating effect of green dynamic capability between green value creation and dimensions of firm performance.

Variables	Environmental Performance		Economic Performance		Customer Value	
	Coeff	95% CI	Coeff	95% CI	Coeff	95% CI
Constants	3.976***	[3.522,4.430]	4.284***	[3.878,4.690]	3.779***	[3.364,4.193]
Business Age	-0.002	[-0.111,0.107]	-0.040	[-0.137,0.058]	0.029	[-0.070,0.129]
Enterprise size	-0.107*	[-0.207,-0.008]	-0.039	[-0.128,0.050]	-0.122**	[-0.212,-0.031]
Nature of business	-0.114	[-0.259,0.031]	-0.269***	[-0.399,-0.139]	-0.077	[-0.210,0.055]
Industry of the company	0.003	[-0.081,0.087]	-0.016	[-0.091,0.059]	-0.002	[-0.078,0.075]
Green Value Creation	0.399***	[0.284,0.515]	0.462***	[0.359,0.566]	0.519***	[0.413,0.624]
Green Dynamic Capability	0.131	[-0.026,0.289]	0.016	[-0.125,0.157]	0.220**	[0.076,0.363]
Green Value Creation* Green Dynamic Capability	0.187**	[0.050,0.325]	0.289***	[0.166,0.412]	0.405***	[0.279,0.530]
R2	0.262		0.377		0.457	0.414
F	12.907***		22.041***		30.651***	25.686

Note: N = 263

* indicates P<0.05

** indicates P<0.01

*** indicates P<0.001

As seen in Table 18, in the model with green value creation, green dynamic capability, and the interaction term between green value creation and green dynamic capability as independent variables and environmental performance as dependent variable, the coefficient of the interaction term between green value creation and green dynamic capability is positive, and its 95% confidence interval is [0.050,0.325] does not contain 0. This indicates that the interaction term between green value creation and green dynamic capability has a significant positive effect on (β = 0.187, p<0.01), which tentatively indicates that there is a positive moderating effect of green dynamic capability between green value creation and environmental performance, and hypothesis H3a is supported.

As seen in Table 18, in the model with green value creation, green dynamic capability, and the interaction term between green value creation and green dynamic capability as independent variables and economic performance as dependent variable, the coefficient of the interaction term between green value creation and green dynamic capability is positive, and its 95% confidence interval is [0.166,0.412] does not contain 0. This indicates that the interaction term between green value creation and green dynamic capability has a significant positive effect on This indicates that the interaction term between green value creation and green dynamic capability has a significant positive effect on economic performance (β = 0.289, p<0.001), which tentatively suggests that there is a positive moderating effect of green dynamic capability between green value creation and economic performance and hypothesis H3b is supported.

As seen in Table 18, in the model with green value creation, green dynamic capability, and the interaction term of green value creation and green dynamic capability as independent variables and customer value as dependent variable, the coefficient of the interaction term of green value creation and green dynamic capability is positive. Its 95% confidence interval is [0.279,0.530] does not contain 0. This indicates that the interaction term of green value creation and green dynamic capability has a significant positive effect on This indicates that the interaction term between green value creation and green dynamic capability has a significant positive effect on customer value (β = 0.405, p<0.001), which initially indicates that there is a positive moderating effect of green dynamic capability between green value creation and customer value, and hypothesis H3c is supported.

**Fig 3:**
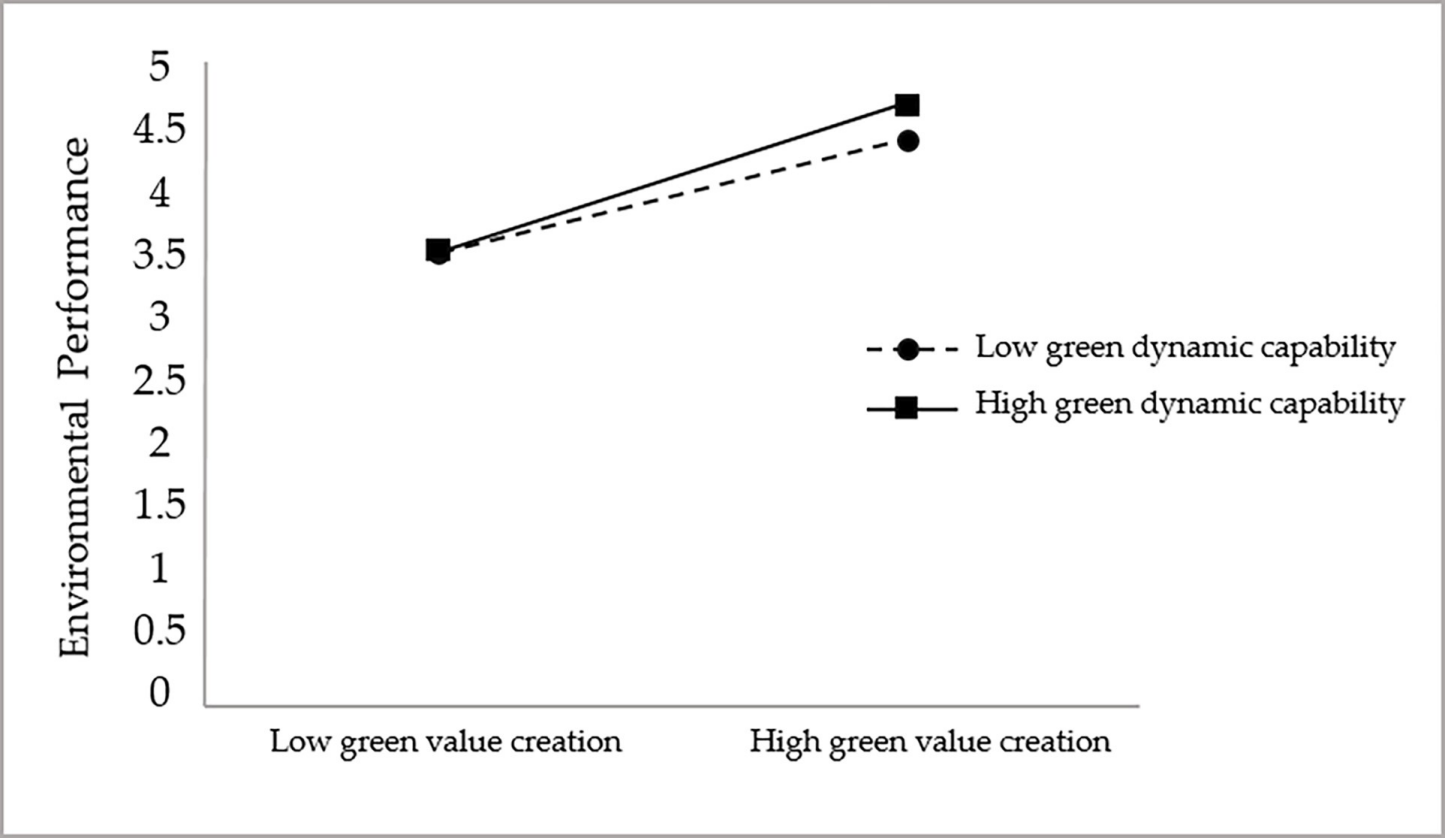
The moderating effect of green dynamic capability on the relationship between green value creation and environmental performance.

**Fig 4:**
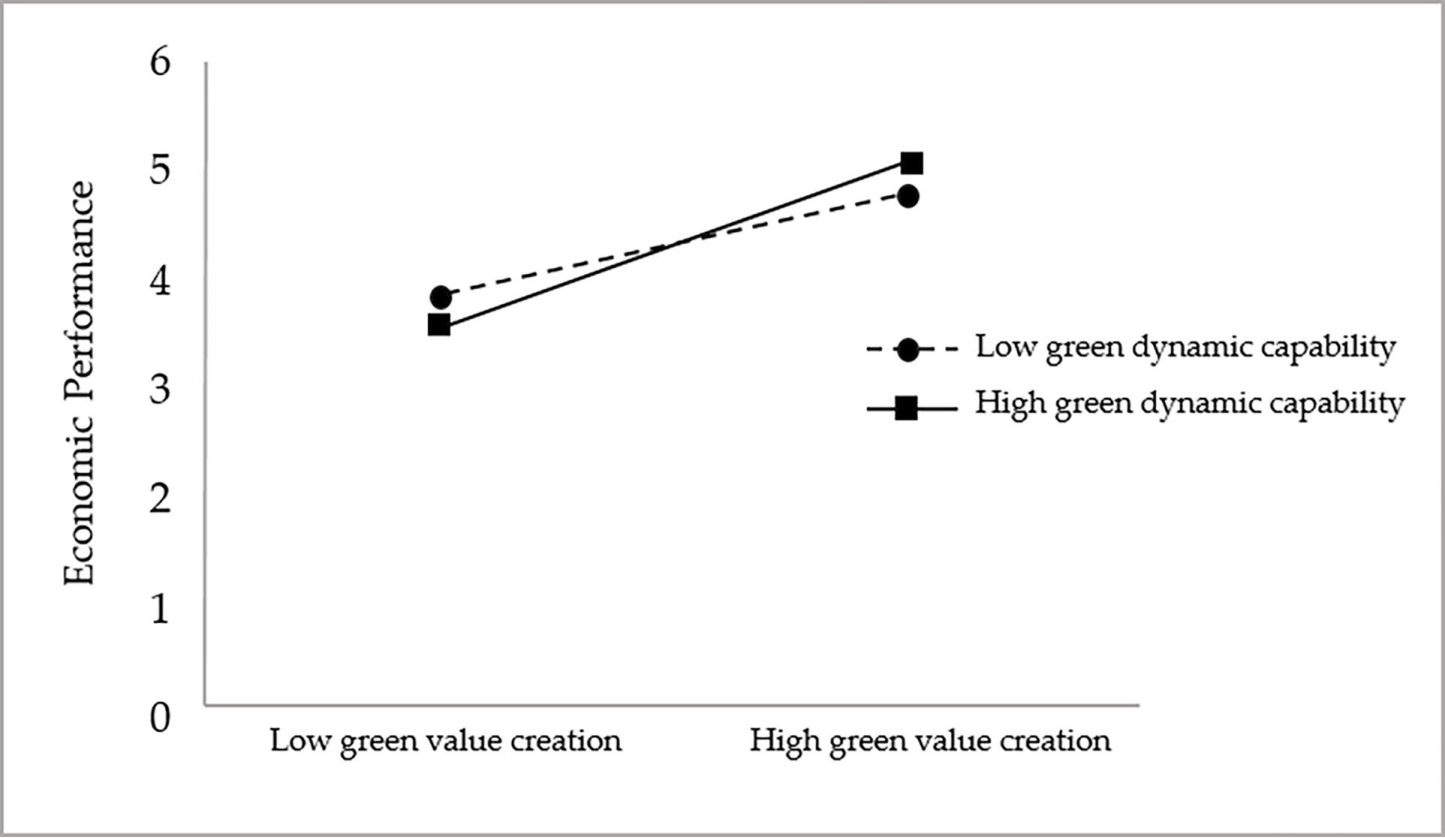
The moderating effect of green dynamic capability on the relationship between green value creation and economic performance.

**Fig 5:**
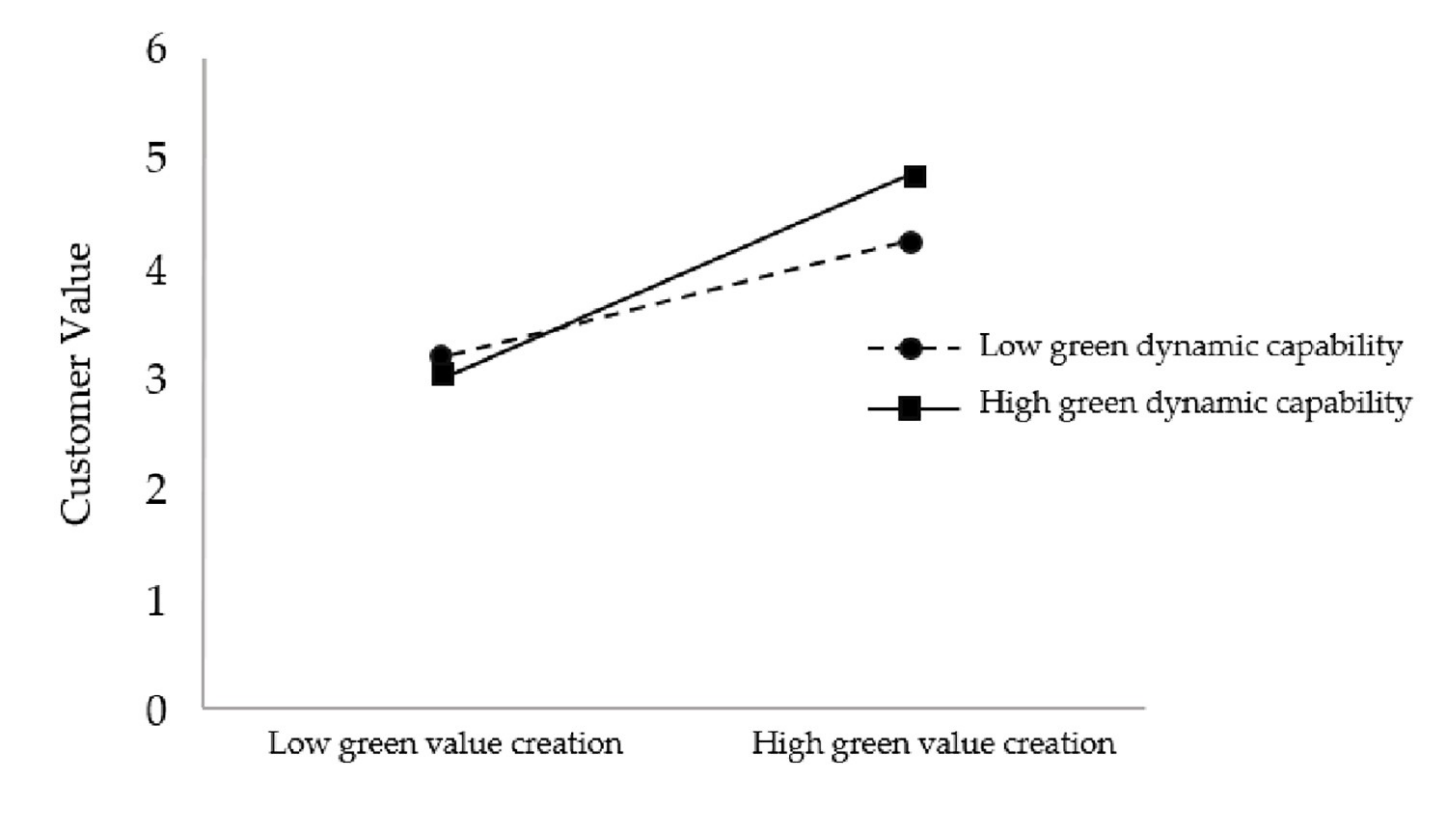
The moderating effect of green dynamic capability on the relationship between green value creation and customer value.

To more visually and clearly show the moderating effect of green dynamic capabilities in the three dimensions of green value creation and firm performance and to further test hypotheses H3a, H3b, and H3c, this study draws the moderating effect diagrams, as shown in Figs 3–5.

As seen in Fig 3, the slope under high green dynamic capacity is greater than that under low green dynamic capacity, thus indicating that the positive relationship between green value creation and environmental performance is stronger under high green dynamic capacity compared to low green dynamic capacity, and hypothesis H3a is further supported.

As seen in Fig 4, the slope under high green dynamic capacity is greater than that under low green dynamic capacity, thus indicating that the positive relationship between green value creation and economic performance is stronger under high green dynamic capacity compared to low green dynamic capacity, and hypothesis H3b is further supported.

As seen in Fig 5, the slope under high green dynamic capability is greater than that under low green dynamic capability, thus indicating that the positive relationship between green value creation and customer value is stronger under high green dynamic capability compared to low green dynamic capability, and hypothesis H3c is further supported.

#### (3) Test of the moderating effect of green dynamic capability on the relationship between green value creation and profit model

The study hypothesized that green dynamic capability plays a moderating role between green value creation and profitability model (H4), and to test this hypothesis, this study used model 1 in Process to test the moderating effect, and the test results are shown in Table 19.

**Table 19:** Test of the moderating effect of green dynamic capability between green value creation and profitability model.

Variables	Profit Model	
	Coeff	95% CI
Constants	4.574***	[4.147,5.000]
Business Age	-0.053	[-0.155,0.049]
Enterprise size	-0.110*	[-0.203,-0.017]
Nature of business	-0.181**	[-0.318,-0.045]
Industry of the company	-0.073	[-0.152,0.006]
Green Value Creation	0.489***	[0.381,0.597]
Green Dynamic Capability	0.279***	[0.131,0.427]
Green Value Creation* Green Dynamic Capability	0.337***	[0.208,0.466]
R2	0.439	
F	28.479	

Note: N = 263

* indicates P<0.05

** indicates P<0.01

*** indicates P<0.001

As seen in Table 19, in the model with green value creation, green dynamic capability, and the interaction term between green value creation and green dynamic capability as independent variables and profitability model as dependent variable, the coefficient of the interaction term between green value creation and green dynamic capability is positive with a 95% confidence interval of [0.208,0.466] not containing 0, thus indicating that the interaction term between green value creation and green dynamic capability has a significant positive effect on In addition, green value creation also has a significant positive effect on profitability model (β = 0.489, p<0.001, 95% CI = [0.381,0.597]), which indicates that green dynamic capability has a positive moderating effect between green value creation and profitability model. Hypothesis H4 was initially supported.

**Fig 6:**
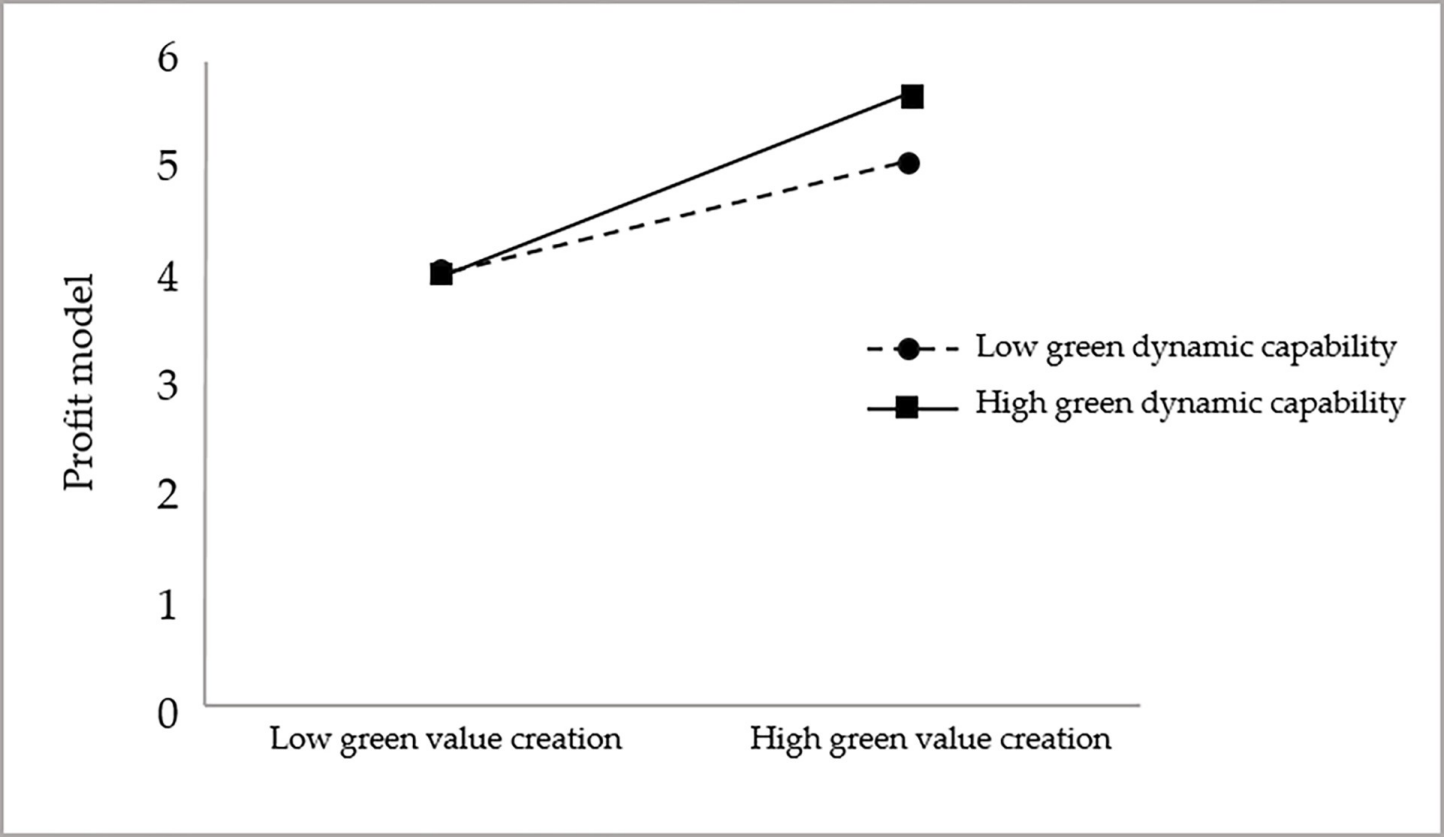
The moderating effect of green dynamic capability on the relationship between green value creation and profit model.

To more visually and clearly show the moderating effect of green dynamic capability in the relationship between green value creation and profit model and further test hypothesis H4, this study draws a moderating effect diagram, as shown in Fig 6.

As seen in Fig 6, the slope under high green dynamic capacity is greater than that under low green dynamic capacity, thus indicating that the positive relationship between green value creation and profitability model is stronger under high green dynamic capacity compared to low green dynamic capacity, and hypothesis H4 is further supported.

## 5 Research results and discussion

A total of 13 hypotheses were proposed in this study, and the results of testing the proposed hypotheses through the analysis of the data are shown in Table 20.

**Table 20:** Summary of study hypothesis testing results.

Assumptions	Hypothetical content	Test results
H1	Green value creation in manufacturing companies significantly and positively affects corporate performance	Established
H1a	Green value creation in manufacturing companies significantly and positively affects environmental performance	Established
H1b	Green value creation in manufacturing companies significantly and positively affects economic performance	Established
H1c	Green value creation in manufacturing companies significantly and positively affects customer value	Established
H2	Profit model mediates between green value creation and corporate performance	Established
H2a	Profit model mediates between green value creation and environmental performance	Established
H2b	Profitability models mediate between green value creation and economic performance	Established
H2c	Profit model mediates between green value creation and customer value	Established
H3	Green dynamic capabilities play a positive moderating role between green value creation and corporate performance	Established
H3a	Green dynamic capabilities play a positive moderating role between green value creation and environmental performance	Established
H3b	Green dynamic capabilities play a positive moderating role between green value creation and economic performance	Established
H3c	Green dynamic capability plays a positive moderating role between green value creation and customer value	Established
H4	Green dynamic capabilities play a positive moderating role between green value creation and profitability models	Established

By empirically testing the green value capture hypothesis model of manufacturing firms, it is shown that green value creation has a significant positive impact on firm performance, profitability model plays a mediating role between green value creation and firm performance, as well as the positive moderating role of green dynamic capability between green value creation and firm performance and between profitability model in the green value capture phase of manufacturing firms.

### 5.1 Theoretical significance

First, to highlight the important role of profit models in the green value acquisition stage of manufacturing enterprises. In discussing the green value acquisition mechanism of manufacturing enterprises, this paper introduces the profit model as an intermediate variable, empirically tests its intermediary effect between green value creation and enterprise performance, and points out that manufacturing enterprises can identify customers’ perceived green value and willingness to pay, design and implement multiple revenue models. Target customers to improve enterprise performance through innovative profit models. This provides a new research approach for improving green value acquisition mechanisms in manufacturing firms and improving firm performance, which fills the gaps in research on green value acquisition.

Second, by introducing the theory of green dynamic capability into the research field of green value acquisition mechanism, it is proposed that green dynamic capability can positively strengthen the relationship between green value creation and corporate performance; on the other hand, the positive strengthening of the relationship between green value creation and profit models enhances the mediating role of profit models between green value creation and corporate performance. The importance of the green value creation mechanism has been clarified in theory. The mechanism of multi-channel realization of green value has been clarified by obtaining information, integrating resources, innovating profit models, and improving the efficiency and effectiveness of green value.

### 5.2 Practical implications

The research findings of this article reinforce the view that green value capture is essential for the green development of manufacturing enterprises. Therefore, managers of manufacturing enterprises need to clarify the need for enterprises to innovate profit models and cultivate green dynamic capabilities actively. They should cooperate with stakeholders in green technology, production, and provision of green products or services. They should also tap into customers’ willingness to pay green, improve the green environmental and economic performance of manufacturing enterprises, and identify them as the driving force for green practices in enterprises.

### 5.3 Suggestion

#### (1) Innovative manufacturing enterprise profit model

Manufacturing companies use big data and digital analytics to tap revenue sources to put customers’ green needs, pain points, willingness to pay, etc. The revenue source of the enterprise includes the transaction revenue obtained through the one-time payment from the customer and the recurring revenue from the customer’s continuous payment after obtaining the value proposition and service. Based on customer demand and the direction of the company’s green product services. On the one hand, companies develop and sustain recurring revenue from different customers. This is mainly through providing specific green value propositions and repeatable and continuous use of services. The more green services customers use, the more they pay. On the other hand, companies develop and sustain one-time revenues mainly derived from the one-time ownership of green products sold.

As personalized green products and customer service customization become mainstream in the future, companies need to transfer the one-time ownership of selling green products and provide services based on green products, thus forming a sustainable revenue model. With the mutual integration of information, digital, and green technology, traditional green products are increasingly developed in the direction of intelligent interconnection. The product’s core value is not the physical product itself but the software that carries it, as well as the services related to the hardware and software products. For this reason, the profit model of manufacturing companies also needs to change from a one-time transfer of ownership to a free + future payment method. For example, Xerox, Xiaomi, and other companies use free or low-priced products to match subsequent continuous services to achieve continuous value acquisition. In other words, they use free or low-cost products to attract customers and use them as the medium to continuously provide related products or services, creating a misaligned profit model and obtaining small but continuous revenue in large quantities.

In addition, diversification of access to value channels. Precise user selection, pricing schemes, and multiple consumption methods, such as remote green value and technology services. For example, through the editable nature of digital technology, we can reduce the cost of value acquisition, provide diversified value supply channels, and expand the revenue model of enterprises.

#### (2) Enhance the green dynamic capability of manufacturing enterprises

Green dynamic capability plays a positive role in regulating the relationship between green value creation and corporate performance. The stronger the green dynamic capability, the lower the loss in the process of converting green value creation into corporate performance, and the more it can meet the customer’s demand willingness, payment willingness, and satisfaction in the process of use, which will lock the customer and form the next virtuous cycle.

Green dynamic capability plays a positive role in regulating the relationship between green value creation and the profit model of an enterprise. The stronger the green dynamic capability, the better the company can collect, analyze, and mine the data related to customer needs, and the more capable it is to change the profit model and promote the realization of customer value as well as the realization of the incentive of stakeholder cooperation.

The green dynamic capability of manufacturing enterprises improves the probability of successful green value creation. It reduces the cost of green value creation activities by deploying heterogeneous resources and technologies, which drives enterprises to propose green strategies. The process of green strategy implementation brings into play the synergistic effect of resources among multiple subjects of green value creation in green R&D, green production, green marketing, green acquisition, etc. It brings political, social, and market resources for the enterprise, enhances the green image of the enterprise, improves the satisfaction of stakeholders, provides a solid foundation for mutual trust and cooperation between the enterprise and stakeholders, and then wins the advantage of green synergistic development, and finally improves the environmental performance and economic performance of the enterprise.

Therefore, manufacturing enterprises should pay attention to the cultivation and enhancement of green dynamic capabilities, make it a long-term strategic priority for the organization, and continuously cultivate and enhance it in the process of continuous learning and innovation, and also focus on cultivating and enhancing the green environmental adaptability and strategic flexibility of the enterprise to cope with the complex external environment. Specifically, manufacturing enterprises take the initiative to establish close cooperation with research institutes and industry-leading enterprises, etc., and through continuous search and excavation of valuable heterogeneous resources, track industry development trends and technology frontiers, focus on the use of new green knowledge while actively investing in green innovative R&D activities, and strive to promote the construction of energy-saving and emission reduction projects and the application of new green technologies and techniques. In addition, enterprises should also create a good innovation atmosphere within the organization, develop various incentive measures to fully mobilize the enthusiasm of staff innovation, and take cultivating and improving the organization’s green dynamic capability as a long-term strategic task to promote green innovation and sustainable development.

In practice, it can build intra- and inter-organizational green learning platforms, improve knowledge transfer and docking mechanisms, fully use external knowledge sources, and continuously adjust and optimize internal resource allocation. At the same time, pay close attention to market opportunities and innovation opportunities and respond to stakeholders’ demands and customers’ potential needs promptly so that the organization’s green learning ability, green resource integration ability, green relationship ability, and environmental adaptation ability can be improved continuously and finally enhance enterprise performance.

### 5.4 Contribution

The contribution of this article lies in the following three points:

First, relevant research on the mechanism of green value acquisition has been expanded in the literature. Early scholars explored the relationship between value proposition, value creation, and value acquisition from a business model perspective. However, there is a lack of attention to the relationship between green value creation and green value acquisition. This paper empirically explores the relationship between green value creation and firm performance, which has important theoretical implications for further research on green value acquisition in manufacturing firms.

Secondly, by proposing and testing the mediating role of profit models. This article explains the inherent logic of the role of green value creation in manufacturing firms in improving firm performance. Previous research has focused more on the impact of green practice activities on firm performance and has also examined the transmission pathways of variables such as sustainable competitive advantage and green innovation between green responsibility and firm performance. However, more attention should be paid to creating and acquiring green value. Therefore, the introduction of profit models as a transmission path provides practical guidance for companies to innovate profit models and create diverse revenue streams. At the same time, it also enriches relevant theoretical research on green value creation.

Thirdly, the regulatory effect of green dynamic capability was introduced and verified. This article shows that at the stage of green value acquisition for manufacturing enterprises, the achievement of green and economic performance depends not only on the number of products and services provided by the enterprise to meet customers’ demands but also on how the enterprise can quickly seize green opportunities, allocate resources, accurately improve customers’ willingness to pay, and promote the transformation of diversified income sources. Therefore, this article incorporates green dynamic capabilities into the research framework and expands the application field of green dynamic capabilities, that is, expands the field of green value creation from green R&D and green production processes and further examines the regulatory effect of green dynamic capabilities on enterprise green value creation.

## 6 Conclusion

Faced with environmental protection constraints and limited resources, how to successfully achieve green development has become a real dilemma for manufacturing enterprises. On the one hand, the green manufacturing technology level of China’s manufacturing enterprises is low, total factor productivity is insufficient, and the environmental performance of enterprises is low; on the other hand, green innovation requires enterprises to invest a lot of preliminary costs, and the government’s environmental regulations will increase the production and operation costs of enterprises in the short term. Some manufacturing enterprises are limited by the cost pressure to actively Some manufacturing enterprises cannot choose green development actively due to cost pressure. Manufacturing enterprises are at the top of the manufacturing industry chain, and the realization of their green value is the new driving force for the high-quality development of the manufacturing industry. It is a meaningful way and means to enhance the core competitiveness of manufacturing. Green development of manufacturing enterprises not only requires effective communication among all departments within the enterprise with value creation as the core, but also needs to consider the green value creation of upstream manufacturers, downstream customers, and other stakeholders to realize the optimization of economic, social and environmental benefits of manufacturing enterprises. Therefore, based on China’s double carbon strategic goal, it is urgent to explore how China’s manufacturing enterprises can collaborate with internal and external resources to realize the green value of China’s manufacturing enterprises.

This paper constructs the role model of green value creation and green value acquisition, the mediating role of the profitability model, and the moderating role of green dynamic capability. The mechanism of action is analyzed empirically using regression methods, and the results of the empirical tests are discussed. The results of this study are as follows. Green value creation positively affects firm performance in the green value capture mechanism. Green value creation significantly and positively affects firm performance, profitability model plays a mediating role between green value creation and firm performance, green dynamic capability plays a positive moderating role between green value creation and firm performance, and green dynamic capability plays a positive moderating role between green value creation and profitability model.

## 7 Study limitation and future research

1. The study primarily relies on questionnaires to gather data for empirical analysis. However, it needs more support of actual case studies from manufacturing enterprises. Future research will supplement the questionnaire data by collecting public data from listed manufacturing enterprises and combining it with case studies.
2. The study focuses on the mediating role of the profit model and the moderating role of green dynamic capabilities in the process of green value capture by manufacturing firms. However, it neglects to consider the impact of government environmental regulations on value capture. Government regulations can influence customers’ willingness to demand green products or services and their willingness to pay for them, thereby affecting green value capture by manufacturing firms. Future research will, therefore, explore the moderating role of government regulation in this process.
